# Teleworking Effects on Mental Health—A Systematic Review and a Research Agenda

**DOI:** 10.3390/ijerph21030243

**Published:** 2024-02-20

**Authors:** Elisabeth Figueiredo, Clara Margaça, Brizeida Hernández-Sánchez, José Carlos Sánchez-García

**Affiliations:** 1 Department of Social Psychology and Anthropology, Faculty of Psychology, University of Salamanca, Avenida de la Merced, 109, 37005 Salamanca, Spain; efigueiredo@usal.es (E.F.); jsanchez@usal.es (J.C.S.-G.); 2 Department of Psychology, University of Valladolid, C/Plaza de Santa Cruz, 8, 47002 Valladolid, Spain; brizeida@uva.es

**Keywords:** teleworking, mental health, workplace, COVID-19, systematic literature review

## Abstract

Teleworking has become an increasingly adopted modality in organizations. However, changes in working conditions have led to several challenges regarding its impacts on professionals’ health. The aim of this study is to provide a systematic review of the literature about the impact of teleworking on workers’ mental health. The PRISMA protocol and VOSviewer were used to identify the main trends from the set of 64 articles. The co-occurrence analyzes showed combined relationships between this new type of work and its effects on workers’ health, which resulted in four different clusters and a robust knowledge structure. Furthermore, the findings indicate that working from home has a dualistic nature. This study offers a prominent and promising framework regarding the teleworking impact on workers’ health research agenda.

## 1. Introduction

After the outbreak of the coronavirus in March 2020, teleworking has become a common practice in all societies around the world [1]. Currently, evidence of the features of this new way of working points to an increasing number of companies that, driven by the search for more efficient teams and work processes, performance, and productivity, have chosen to put their employees to work from home [2,3,4]. According to recent statistical data from the International Labor Organization [5], in the European Union, an average of 3 out of 10 employees work remotely and assume, autonomously, their professional activity, managing time, and execution of tasks.

Telework is defined as a work pattern that implies that employees perform their functions outside the company to which they are contractually bound using technological and digital tools and equipment [5]. This recurrent organization of work, which influences management strategies and company culture, is recognized for offering advantages for both companies and employees [3,6,7,8], among them: reducing the time and costs associated with travel to the place of work, greater flexibility in working hours [9,10], autonomy in the management and execution of tasks [11,12], increased quality and efficiency of production, greater security, and better possibilities for articulating professional and personal life [6,13]. Several studies, e.g., [14,15], have shown that teleworking induces a greater level of satisfaction in employees when compared to individuals who do not practice this modality of work.

It can be assumed that the 2000s were the heyday of new technologies and the internet, which aroused interest in new forms of work organization. In recent years, teleworking has gained prominence in all sectors, driven by the pandemic crisis, and, according to Thevenon [1], a gradual growth of this labor phenomenon is expected. However, despite the numerous benefits, e.g., [6,16], working remotely poses challenges for employees and may even pose a risk to the physical and mental health of professionals [3,17,18]. Changes in working conditions [19,20], isolation, monotony due to lack of coexistence and face-to-face interaction [21,22,23], the feeling of being forgotten by the company, career stagnation, and imbalance in the management of personal life with professionals represent strong constraints, which are identified as being at the origin of the many negative consequences for the psychological health of workers [24,25]. In this sense, these difficulties often faced by teleworkers regarding technical problems (e.g., internet connection, access to company servers, or ergonomic conditions) can contribute to situations of anxiety, stress, and emotional exhaustion [17,26,27]. In view of this, the literature points to a substantial deterioration in the mental health of remote workers, which represents the starting point for the development of professional illnesses, such as anxiety, stress, depression, lack of self-esteem, insecurity, psychosomatic problems, and exhaustion [17,24,28,29]. Several studies carried out in previous, during, and post-pandemic periods highlight the moderating effect that remote working conditions can have on the psychological health of the workers, e.g., [15,30].

The literature on mental health in the work context addresses issues related to emotional, psychological, and social well-being [16], which allows individuals to face the demands of daily life in a balanced and productive way, using their emotive and cognitive faculties, exploring potential, and contributing to the community. For Shipman et al. [31], mental health and human performance are not two distinct parts but are, in essence, one and the same, which fosters the development and full realization of the individual. For instance, the isolation that comes from remote work entails psychological, emotional, and social challenges [32,33], which can lead to the development of depressive behaviors, significantly compromising satisfaction and the quality of life at work [17,25,34]. For instance, burnout, widely known in the world of work, results from a permanent state of emotional, mental, and physical exhaustion [17,35,36]. Adaptation difficulties in different organizational contexts or due to the implementation of new work methodologies may be at the origin of the increase in burnout situations reported by remote workers [35]. According to Meyer et al. [37], there is a positive association between working remotely and the level of professional burnout.

The number of individuals opting for telecommuting has been growing rapidly [1,38]. In this sense, the study of the psychological impacts of this new work reality becomes urgent. To increase awareness on these issues and point out the current perspectives within this research field, the following research questions were raised:

RQ1. How does teleworking influence mental health and how this is expressed in the literature?
RQ2. What effects did the COVID-19 pandemic have on teleworkers’ mental health?
RQ3. What is the typology of the symptoms associated with remote work?
RQ4. How did the knowledge structure of teleworking and mental health evolve over time?

In view of the above, this study will be of particular relevance for companies and may contribute to the definition of more effective strategies for implementing telework and mitigating the risks associated with this type of work. This article is organized as follows:

## 2. Materials and Methods

### 2.1. Inclusion Criteria and Analysis

The Scopus database was used, due to it being the most recommended, especially because it covers the Social Science Citation Index (SSCI), which is included in the Web of Science database [39]. In addition, taking the search protocols as a reference, the following search criteria and Boolean codes were used: “telework* OR telecommut* AND mental health”. Mental health is understood as a state of mental well-being that allows people to deal with stress, work well, sustain capabilities, and make decisions. Furthermore, the concept of mental health encompasses emotional, psychological, and social well-being. For this reason, to achieve a greater scope of the different underlying symptoms, only the concept of “mental health” was searched. To avoid bias, the dual review [40] method was used. Separately, two authors analyzed the set of selected articles for subsequent comparison and discussion, seeking consensus. Whenever there was any disagreement, assistance was requested from the other two authors for discussion and evaluation.

The guidelines by Busenitz et al. [41] and Kraus et al. [42] were used. This review only included scientific articles (excluding books, book chapters, and conference proceedings) in order to comply with the theoretical and methodological principles. A set of scientific articles written in English, Portuguese, Spanish, and French were analyzed since the authors of this study master the four languages. As the first step, each author carefully read the title and abstract and, whenever any doubt arose, the complete article. Secondly, the authors analyzed their selection, and, in case of disagreement, the article was analyzed repeatedly until reaching a consensus to include or exclude it. Finally, after this initial process, 44 articles were excluded for two reasons ^1^: 1. they did not refer to telework but to work in general; and 2. mental health issues were not the main focus of the studies. A final sample of 64 articles was obtained.

**Figure 1:**
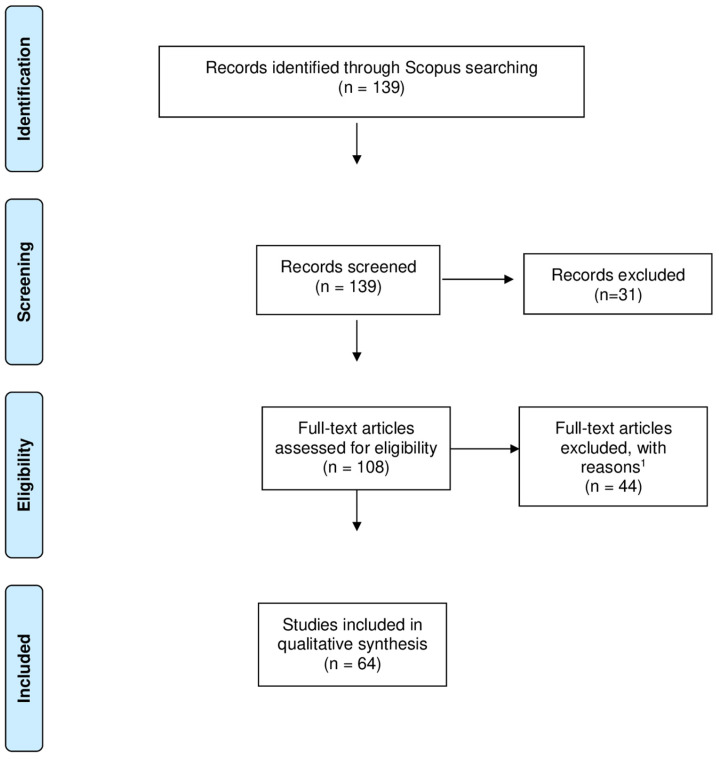
PRISMA flow diagram.

A thorough search was carried out in a comprehensive and reliable database and, for this purpose, the PRISMA (Preferred Reporting Items for Systematic Reviews and Meta-analysis) protocol was used in this systematic review, as can be seen in Figure 1. This guide enables the replication of results, focusing on their clarity, transparency, and coherence [43], in addition to contributing to the reduction of biased processes [44]. VOSviewer 1.6.10 software [45] was used to perform visualization analyzes and create bibliometric maps [46]. Namely, the analysis of the co-occurrence of keywords makes use of frequent terms, which allow the identification of current themes on the subject under analysis [47].

### 2.2. Descriptive Analysis

**Figure 2:**
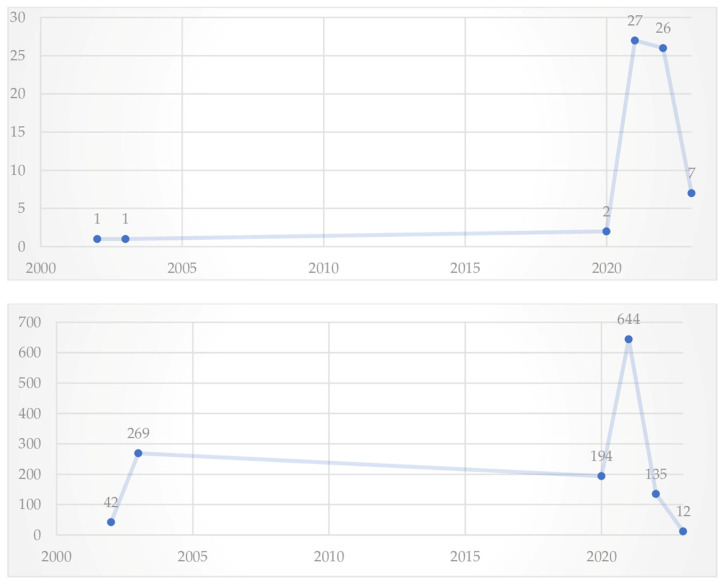
Number of articles and citations by year.

The evolution of publications and citations each year on teleworking and mental health is illustrated in Figure 2. This theme emerged in 2002 and showed an exponential growth in 2020. Only in the early 2000s were publications presented inconsistently. In the year 2021, there was a growing trend of publications, a fact that can be largely justified by the reality of teleworking driven by the lockdown. With regard to citations, it is possible to observe that the year 2021 obtained 645 publications, as much as the sum of the years with publications.

The top three articles with the most citations are:

1. Mann and Holdsworth, 2003 [21]: 269 citations;
2. Xiao et al., 2021 [48]: 237 citations;
3. Oakman et al., 2020 [49]; 188 citations.

**Figure 3:**
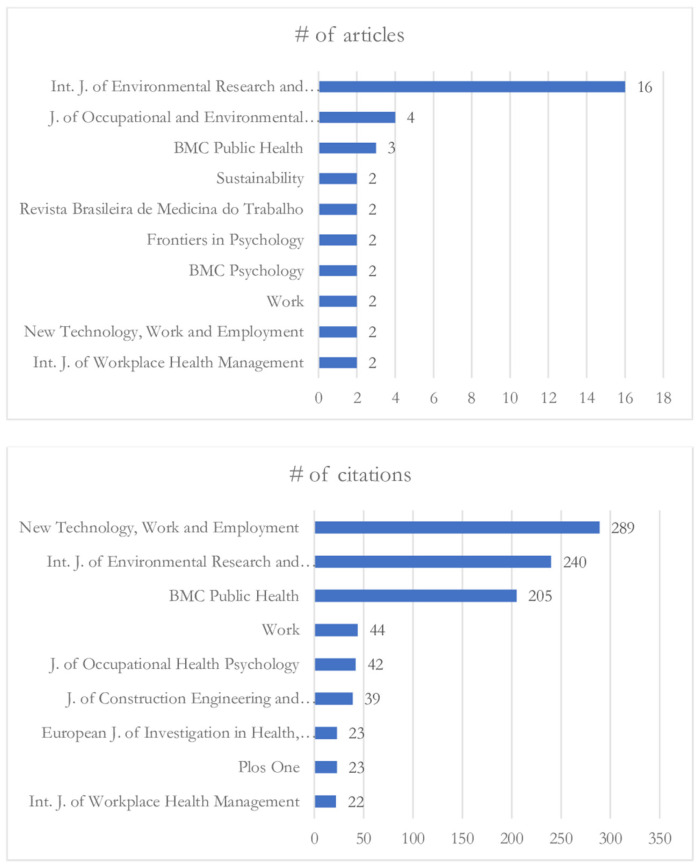
Distribution of articles and total citations by journal.

Figure 3 shows the ten journals with the highest number of publications, as well as the number of citations. Those with three or more articles are: the *International Journal of Environmental Research and Public Health* (16 articles), *Journal of Occupational and Environmental Medicine* (4 articles), and *BMC Public Health* (3 articles). The journals with the most citations are: *New Technology, Work and Employment* (289 citations), *Journal of Occupational and Environmental Medicine* (241 citations), *International Journal of Environmental Research and Public Health* (240 citations), and *BMC Public Health* (205 citations).

These 64 articles have a total of 273 authors, which makes an average of 4.26 authors per article. The percentage of authors who published more than one article on the subject is residual (5.49%).

**Figure 4:**
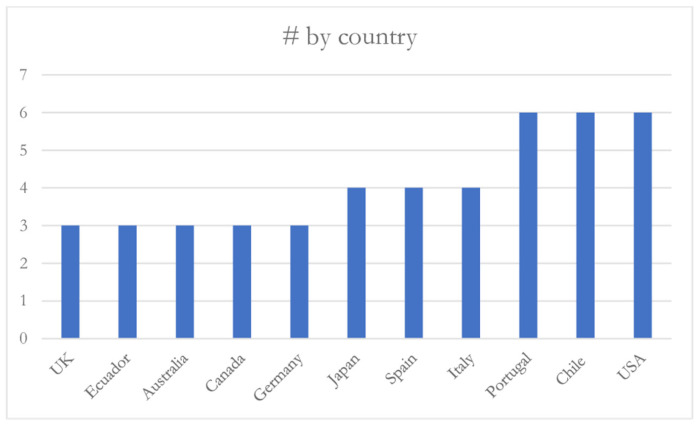
Geographic distribution.

Regarding the geographical distribution of these publications, Figure 4 shows where 70.03% of the articles were published. The remaining publications are geographically dispersed: France, Sweden, Colombia, and Brazil, with two articles each, and Mexico, the Netherlands, Argentina, Latvia, South Korea, Lithuania, Poland, Oman, Indonesia, Malaysia, and Turkey with one article each.

The analyzes referring to the methodology and the type of sample can be analyzed in Table 1. It is clear that most of the studies are of a quantitative nature (71.8%) and applied to generalized workers.

Finally, the main supporting theories were the Conservation of Resources Theory (5 articles) and the Job Demands–Resources Theory (4 articles). The Conservation of Resources Theory analyzes psychological stress and studies its nature, which can have environmental, social, and likely consequences. Furthermore, it is particularly useful for understanding the relationships between stress and physical health, as well as the effects of occupational burnout [50]. Briefly, the Job Demands–Resources Theory proposed by Bakker and Demerouti [51] is a model of occupational stress that suggests that tension is a response to the imbalance between the individual’s demands and the resources they have to deal with these demands, regardless of the work sector.

### 2.3. Cluster Analysis

**Figure 5:**
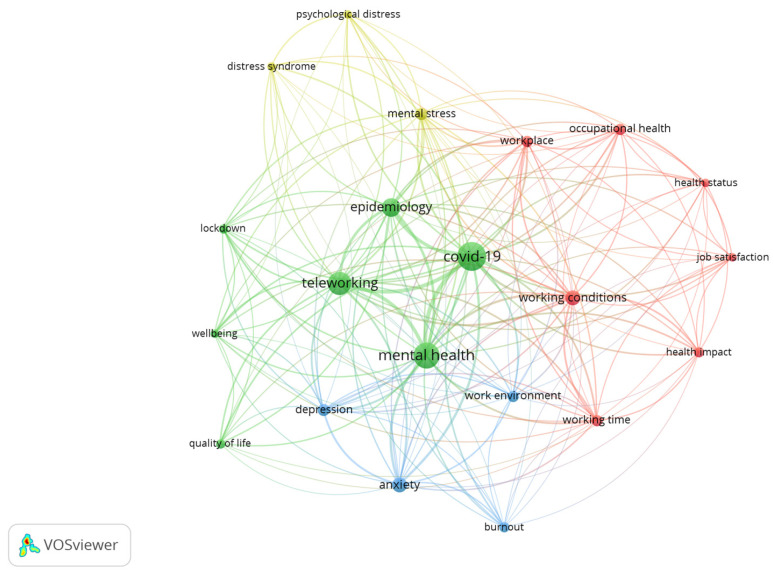
Five co-occurrences for keywords. Source: VOSviewer version 1.6.10.

Within the scope of the study carried out, the identification and analysis of keyword associations play a fundamental role, as they enable a more detailed and comprehensive understanding of trends in the corpus of publications. Figure 5 illustrates a visual map, a set of graphic networks, marked by different colors (green, blue, red, and yellow) [47]. The different colors reflect the existence of 4 clusters, interconnected and overlapping, which are described in detail in Table 2.

#### 2.3.1. Cluster 1—Work Effects on Health

The first cluster, in red, includes a set of 7 keywords, centered on work effects on health: health impact, health status, job satisfaction, occupational health, working conditions, working time, and workplace, corresponding to approximately 40% of occurrences.

In this cluster, the greatest emphasis is given to the working conditions. This concept is intrinsically related to the work environment, job satisfaction, and occupational health. In the business context, working conditions are a key factor that directly influences workers’ occupational health [55], and, consequently, job satisfaction is closely linked to these two factors. Several studies grouped in this cluster analyzed the effects of working conditions on an individual’s health, which reflects the relevance of this topic in the modern working world. Considering the fact that people spend a significant part of their lives in the workplace, the work dimension, which encompasses occupational health, working conditions, working time, and the workplace [30,56], can cause serious impacts on workers’ health, influencing their well-being and satisfaction [11,57]. This finding is corroborated by Perelman et al. [53], when highlighting the relevance of occupational health, particularly in the remote work environment. These authors consider occupational health as a critical condition in promoting individual health and implementing a healthy work environment for teleworkers.

Working conditions encompass several factors, such as the physical environment, available resources, workload, interpersonal relationships, communication, and organizational culture [20,55,57,58]. In this context, a safe work environment, well organized and equipped with the right tools, becomes essential to ensure that workers feel safe and satisfied, as well as working effectively, reducing work-related stress [59]. Flexible working hours and the possibility of working remotely are two complementary variables that can potentially contribute to improving the work environment and increasing employee satisfaction and professional commitment [9,10].

The quality of working conditions and the promotion of health in the workplace are two fundamental pillars, which directly influence the performance of employees and company’s success. These elements, which are interconnected, play a crucial role in the operational efficiency of organizations [57], as they prevent work-related illnesses and guarantee productive working environments. When well-managed, these resources promote a healthy and satisfactory work climate, helping employees feel more valued and motivated [60].

In recent decades, concerns about working conditions and occupational health [7] have gained prominence in academic research, acquiring greater importance during the COVID-19 pandemic, especially in the context of teleworking. For instance, in countries such as Indonesia [12], Germany [30], Chile [61], Ecuador [62], and France [63], it is clear that the work environment has influenced the increase in mental health problems, which is reflected in terms of productivity [64] as well as work quality and performance.

In the context of remote work, some authors concluded that the effects on workers’ mental health may vary, depending on several factors, especially the conditions and demands of the work [48,65]. Consequently, and according to these researchers, remote work leads to exhaustion situations, on the one hand, if workers are not provided with adequate working conditions (e.g., poor ergonomics, inappropriate work surfaces, acoustic problems, and poor infrastructure) [66] and, on the other, when they feel overwhelmed [10] or isolated and without support from managers [54].

Several authors support the need to ensure favorable working conditions for individuals who are working from home, e.g., [62,67]. To this end, according to these authors, it is necessary to promote social interactions and the practice of emotional support as relevant measures to mitigate the adverse effects of isolation on the mental health of teleworkers. The importance of interpersonal relationships and the social environment in the work context is reinforced [20]. The authors argue that, in a remote work context, the absence of physical social interactions can generate a feeling of isolation and profound loneliness [1], which will compromise productivity [64] and the creativity of individuals [12,17,68].

Working from home is described as a potential resource, whose conditions and surroundings can have different effects on the worker’s health [9,12,69,70]. On the one hand, the benefits mentioned by the authors include a greater level of satisfaction and well-being, as well as a better quality of life, which arise due to greater flexibility in working hours and patterns and time savings in daily travel [10]. In addition, a balanced diet and relief from the workplace pressures are also mentioned. On the other hand, regarding the negative effects, mental health and psychological disorders appear to be the most challenging consequences of remote work, with records of a considerable increase in headaches, irritability [61], impatience, anxiety, fear, discouragement [21,69], lack of motivation [23], irregular sleep, general tiredness, and feelings of ineptitude with the work due to essentially working conditions, work intensity, and a substantial increase in the number of hours worked [20,22,30,71,72].

Teleworkers with less support and poor supervision had a higher rate of mental suffering [73,74]. In line with this, other authors highlight the importance of working conditions in promoting the health and mental well-being of teleworkers, with a particular emphasis on social and technical support as crucial success factors [62].

#### 2.3.2. Cluster 2—Pandemic Effects

In general, the publications focused on how the environmental conditions caused by the COVID-19 pandemic forced professionals to readjust their posture and behavior towards work, which led to the reinvention of the way of working [75,76].

The literature presents a wide variety of effects caused by the pandemic on the health and well-being of workers, which were manifested directly and indirectly [60,72]. Thus, cluster 2, in blue, is made up of the following set of keywords: COVID-19, epidemiology, lockdown, mental health, quality of life, teleworking, and well-being. This cluster constitutes one of the main themes of this study, highlighting strong connections with other clusters, which emerge as the most influential domains in our analysis.

Recognizing the COVID-19 pandemic as a phenomenon that catalyzes several changes in the dynamics and work sphere [12,19,20], the articles analyzed suggest scientific evidence regarding the impact of the lockdown and the adopted measures, namely on the imposition of remote work, on the quality of life, and on the mental health status of workers [23,53,65,73,77].

In this context, and given the increase in teleworking in recent years, the focus of studies has also been on the role that the pandemic played in the implementation of teleworking and the several challenges that this new work modality entailed. As an example, a study conducted in 2022 aimed to analyze the effects of the COVID-19 pandemic in relation to the implementation and rise of teleworking [67], whose results highlight the fact that the pandemic significantly increased the adoption of teleworking around the world. This type of finding makes it clear that this work pattern had varied impacts on the well-being and mental health of workers [70,72], noting different effects.

Other studies [72] corroborate these findings, showing that teleworking, during the pandemic period, had an adverse influence on workers’ behavior, which resulted in a substantial increase in cases of anxiety and depression, which also translates into significant lower productivity and performance [12,64]. The health problems potentially associated with remote work during the pandemic were highlighted [78]. Furthermore, it was also possible to assert that women were more affected when compared to men, both on a professional social and personal level [22,55,68,69,71,72,79]. The expectations of managers and the lack of flexibility were the triggers for the increase in stress and feelings of inadequacy at work.

During the pandemic, several researchers, e.g., [19,69,75,80,81], specifically analyzed the challenges faced by teachers when teaching remotely. Distance learning, the modality adopted by most educational institutions around the world in response to the lockdown, brought significant consequences for teachers [58]. For instance, it was found that online teaching had a critical impact on the health of these professionals [74], with a higher incidence of psychiatric episodes, particularly in those who were initially suffering from some health problems [70]. When asked about this aspect, a large percentage of teachers (86%) pointed out the lack of face-to-face interactions with colleagues and students and the work–life balance as two of the most challenging factors of remote work, which resulted in a significant workload, as it required more time and effort than face-to-face teaching. The teachers who suffered the most mentally from teleworking were female teachers [74], particularly those with young children, due to gender inequalities and the roles assumed by each member of the couple in carrying out tasks, e.g., household chores [48,79]. Complementarily, other authors [61] found that, during the confinement period, teachers presented higher levels of mental suffering than other worker groups due to transfer and the need to quickly adapt to new technologies and teaching methodologies.

One year after the pandemic outbreak was declared, stress and burnout continued to affect education professionals, with 72% of teachers declaring that they continued to feel very stressed and 57% very or extremely exhausted [80]. Concern for students, the need to create online content, evaluating students virtually, and the lack of adequate support from educational institutions to deal with issues of this nature were clear reasons that contributed to major moments of stress and various health disorders. By way of conclusion, the mental health of workers must be a fundamental factor in the success of teleworking when implementing it [82]. These authors reiterate that in order to mitigate the adverse impacts of teleworking on mental health, it is essential to provide adequate support and resources to workers who perform their duties remotely.

#### 2.3.3. Cluster 3—Emotional Effects

Cluster 3, in blue, encompasses the keywords anxiety, burnout, depression, and work environment, highlighting the psychological disorders generated by remote work.

With the expansion of remote work following the pandemic, issues related to the mental and psychological health of workers have been increasingly researched. The set of publications that constitute this cluster reveal negative experiences, such as loneliness, irritation, discouragement, and worry, which have been manifested more clearly in teleworkers compared to workers who work in person in offices [21]. On the one hand, distance from colleagues and, on the other hand, in-person work environment contact are clearly the biggest challenges that can lead to feelings of isolation and loneliness, affecting the emotional health of workers. In this context, several articles, e.g., [80,81], highlighted a picture of negative reactions associated with teleworking, with an emphasis on anxiety, burnout, and depression, which inevitably compromise workers’ performance.

In this cluster, it is worth highlighting the studies carried out by [61,62], which show that the COVID-19 pandemic was responsible for generating stressful situations and changes in the structure of working time. In this context, for many teachers and staff, the obligation to continue carrying out educational tasks remotely has triggered situations of decompensation, marked by bouts of anxiety and depression. For instance, for psychologists, teleworking during the pandemic represented a set of challenges and constraints and found significant differences between psychologists who worked in person and those who were teleworking [83]. For these professionals, working from home represented a greater level of personal (34%) and professional (37%) exhaustion and an increase in stress and depression.

Teleworking during confinement contributed to the increase in the prevalence of anxiety symptoms among workers, finding that 32.1% of participants showed signs of anxiety disorder and 7.65% suffered from depressive disorder [63]. Factors such as work overload, changes in work schedules, difficulties related to infrastructure (e.g., poor internet connection), conflict between family and professional life, and disturbances in sleep quality are among the factors that most contributed to the decline in mental wellness [66,69,81].

Pathologies associated with the work environment, such as burnout, arise in response to a set of permanent circumstances, such as emotional exhaustion [11], and mental and physical fatigue, when the individual is exposed to situations of prolonged stress [17,35,36,80]. The individual’s behavior changes, and negative thoughts and feelings of weakness and ineffectiveness regarding work emerge, impacting their performance and productivity [12,64,68]. In other words, exhaustion suggests a negative psychological state, which is generally accompanied by a feeling of frustration and exhaustion, which means that the usual ways of coping are no longer working [84,85]. There is a positive association between working remotely and the level of professional burnout [37]. Hence, the use of the most current digital and information technologies [19], associated with difficulties in adapting to new organizational and technological contexts [81], can substantially provoke burnout situations [35,80]. It was possible to conclude that long-term teleworkers are among the individuals who present the most severe rates of anxiety and depression compared to those who telework for a shorter period, and, of the entire population studied, 40% of teleworkers reported depressive and anxious tendencies as a result of working remotely [77]. The lack of work–life balance is indicated as one of the factors resulting from teleworking, which can trigger burnout because it leads to longer working days [68] and an increase in stress rates [81].

The concept of teleworking is not recent. Mann and Holdsworth [21] already drew attention to the potential emotional impacts of teleworking. These authors highlighted that teleworkers, compared to those who worked in person at a company, experienced higher levels of loneliness resulting from isolation, greater irritability, worry, lack of confidence, sense of guilt, and frustration. In recent years, the literature has been recognizing the challenges posed by this new working methodology. It is undeniable that teleworking causes negative impacts on the various spheres of people’s lives, which is why it is essential to develop strategies to mitigate these scenarios and, in this way, promote a work–life balance.

#### 2.3.4. Cluster 4—Stress and Teleworking

The fourth cluster, in yellow, represents 17% of co-occurrences and encompasses the following keywords: distress syndrome, mental stress, and psychological distress.

Several articles, e.g., [19,56], highlight that teleworking is an increasingly common practice. However, what is also clear are its potential adverse consequences, such as mental exhaustion, suffering, and psychological anguish. Just as evident is the absence of face-to-face social interactions [20,21,22,86] and the difficulty of establishing clear boundaries between professional and personal life, which can contribute to these negative psychological states. For instance, during confinement, teleworking caused mixed effects on the psychological well-being of the Italian academic population; that is, on the one hand, some academic workers reported high levels of satisfaction with teleworking and, on the other hand, others experienced discomfort in their mental health, with pronounced anxiety, depression, and burnout disorders [87]. In line with this, during the lockdown, in France, it was found in a sample of hospital employees that the prevalence of stress syndrome and psychological distress was higher for teleworkers than those who worked in the hospital [63].

An increase in unhealthy behaviors was evident among respondents, who reported higher levels of psychological distress and lower levels of well-being [54]. The authors concluded that there was an increase in alcohol consumption, and smoking participants began to smoke more cigarettes per day. It should be noted that this type of behavior (e.g., drinking alcohol and smoking) is acquired as a way to face and respond to psychological stress [88].

Finally, other authors emphasized that the relationship between teleworking and psychological stress depended on the worker’s position and preferences regarding remote work [56]. The individuals who did not identify with teleworking were more exposed to psychological stress when compared to other workers. For example, a study concluded that remote work can increase and/or intensify levels of psychological stress, especially in situations of uncertainty, such as during the COVID-19 pandemic [67].

## 3. Discussion

This systematic review aimed to analyze a set of carefully selected articles published between 2000 and 2023. Through this bibliometric analysis, the impacts of teleworking on the psychological and mental health of workers were circumscribed with the aim of contributing to a more comprehensive understanding of the phenomenon. To this end, and based on PRISMA guidelines, a set of 64 articles were included in this review, obtained through a structured search on Scopus. For this search, three selection criteria were defined and simultaneously three time periods: pre-pandemic, during pandemic, and post-pandemic caused by COVID-19. Based on the selected publications, a bibliometric analysis was carried out with the aim of exploring the most frequent terms in the literature. For this purpose, VOSviewer software was used, a bibliometric analytical tool used to map the literature and provide visualization through the clustering technique and graphic networks [89,90]. In this sense, the set of articles included in this study allowed us to obtain a complete framework of the existing literature on teleworking and its impacts on mental health.

Firstly, these results indicate that teleworking predates the recent pandemic crisis. However, it might say that this new modality of work is an emerging and current phenomenon, which, by necessity, gained new momentum with COVID-19 [78] and which already allows us to guide an appropriate response to the first research question. From March 2020 onwards, the world was affected by a phenomenon that required adaptation to the numerous restrictions imposed, and the work sphere was no exception [67]. In recent years, this thematic has been the subject of several studies, registering a growing interest among academics regarding the implications of this phenomenon for both organizations and workers.

There is consensus in the literature regarding the fact that COVID-19 has had different impacts on people’s lives, particularly with regard to quality of life, as a result of the different restrictions imposed on social activities. Furthermore, this phenomenon also profoundly changed work dynamics, forcing workers to adapt to a new work context, which was a trigger for cases of health disorders, particularly emotional, psychological, and mental. In this sense, the period during and after the pandemic has been the focus of study regarding the prolonged effects of confinement and teleworking on health [91]. The items analyzed make it clear that, from 2020 onwards, there was a significant increase in scientific production in this area, with 2021 being the year in which there was a greater number of publications.

Of the set of articles analyzed, quantitative studies predominate (71.8%), which were carried out in different countries, and this point must be considered since the divergence observed in some factors such as culture, economy, and society can be decisive in the results presented.

The co-occurrences found allowed the establishment of four major categories or clusters, regrouped by the analysis of their scientific proximity: 1. work effects on health; 2. pandemic effects; 3. emotional effects; and 4. stress effects.

Regarding the set of publications that make up the first cluster, working conditions and the number of working hours were analyzed [48], emphasizing the implications of these factors on workers’ health [22,30]. This cluster also focuses on job satisfaction [52,57,87], not only as a result but also as an element that allows evaluating the state workers’ health and the viability of remote activity. Thus, the results indicate that an approach that prioritizes the empowerment of professionals, recognition and rewards [55], technical and social support, organizational culture [55,57], and communication [10] represents powerful inputs for the satisfaction, health, and well-being of teleworkers [9].

Working conditions and occupational health are essential areas for the sustainability of organizations [7,30,57]. Therefore, these two dimensions should be seen not as mere parameters but rather as fundamental strategic investments in human resources, aiming for long-term business success. In this sense, organizations that prioritize the well-being of their employees are more capable of strategically setting themselves apart from the competition, sustaining the benefits of a healthy and satisfied workforce, which will have positive repercussions on organizational performance [12]. In other words, promoting workers’ well-being is a crucial organizational path, with clear impacts on both individuals and companies, and this must be part of an effective and sustainable business management policy.

The detailed analysis of cluster 2 sheds light on the answer to the second research question, as this cluster focuses on the effects resulting from the COVID-19 pandemic, particularly in work-related areas, quality of life, and individuals’ well-being and mental health. There is a consensus that the lockdown and the transition to teleworking had a significant impact on people’s daily routine [60]. This ‘new normal’ generated feelings of fear, uncertainty, and panic [69]. This new style of work began to represent a radical change in the way people began to carry out their work activities [11,19,20], which led to a significant increase in mental disorders (e.g., anxiety, burnout, and depression) among workers [23,68]. The inevitable social isolation during this period, due to the lack of socialization with co-workers, increased cases of anguish and psychological suffering among teleworkers [24,31]. Clusters 3 and 4 bring together studies that focus on the emotional and stress effects resulting from the new work context, as well as the main associated pathologies: burnout, anxiety, mental stress, psychological suffering, and other mental disorders.

As research into teleworking becomes more galvanized, it becomes clear that this new work paradigm entails a variety of physical, emotional, and social symptoms, which can affect the quality of life, health, and performance of workers [76,92]. Regarding the third research question, we can divide the symptoms into two large groups: (1) ergonomic and musculoskeletal, essentially due to the lack of adequate ergonomic conditions and a sedentary lifestyle; and (2) psychological symptoms, such as anxiety, stress, and anguish, closely related to the lack of work–life balance. It is worth highlighting the emotional and mental health spheres, namely burnout and prolonged isolation. Subsequently, it is important to mention that this last group of symptoms (e.g., stress, anxiety, and depression) can worsen or accelerate the progression of physical illnesses.

To answer the fourth research question, it should be noted that working from home before 2020 (that is, before the outbreak of the COVID-19 pandemic) was considered a luxury. For instance, only 7% of the world’s population worked from home before the pandemic, according to statistics from the International Labor Organization [93]. In contrast, in 2022, 16% of jobs were 100% remote. Alongside this global phenomenon, the advancement of digitalization and technology and the shift to a knowledge-based economy have made the option of remote work possible for employers and employees. In this sense, the ability to work remotely has become crucially important for work and organizational decisions. By interconnecting teleworking with mental health, it is possible to assert that we have moved from a superficial understanding to a more holistic approach, which not only highlights the challenges but also defines strategies with a view to mitigating adverse effects on workers’ health and promoting a healthier and more productive work environment. Given the intensification of teleworking in today’s society, the psychological health of workers has become a concern for managers.

On the one hand, teleworking is cited as a source of well-being, quality of life, and job satisfaction [57,60]. However, on the other hand, this type of work can also have adverse effects on the psychological and mental health of individuals [3,94]. The literature highlights that working from home can quickly become critical for many workers, who can develop emotional, psychological, and mental problems [18,31,33]. The results indicate that teleworking can influence workers’ behavior, regardless of the services or positions held (e.g., teaching, administrative, psychologists, and hospital staff). That is, irrespective of the role performed, workers are exposed to a set of factors that can trigger different pathologies, a finding found mainly among female workers [9,19,22,55,68,69,71,78,79,81].

In general, teleworking, intensified by the lockdown, led to an increase in stress, anxiety, and depression among workers [83], also affecting their mood [82], generating emotional exhaustion. This altered health situation can lead to bouts of exhaustion, low self-esteem, and insecurity [31]. Furthermore, this situation can potentially be worsened by a set of factors, such as social isolation [32,95], limited mobility [56], poor working conditions [58], number of hours worked [52,68], lack of communication [9], and the absence of support from colleagues and supervisors. These conditions, which can be perceived separately or together, represent an imbalance between work requirements and the physical and mental capabilities of workers, which can accentuate feelings of frustration and inertia [49,96]. Furthermore, the lack of delimitation between personal and professional life, noted in many contexts [71,81], can increase the risk of exhaustion/burnout and work overload [10,66].

Among the many other critical factors that predispose workers to mental health disorders, the literature highlights the perception of a changed relationship with the hierarchy, the difficulty in establishing virtual communication [56], the perception of support organizational deficiencies [97], a lack of recognition [55] and reward policies, a lack of feedback [98], and feelings of no longer belonging to the company [29]. Also, the partial or total absence of these factors contributes to the development of exhaustion [31], disinterest [32], and little involvement with the organization and work [26]. Furthermore, these factors can trigger the emergence of psychosomatic disorders such as sleep problems [69], increased alcohol consumption, and smoking [54]. The difficulty in adequately managing this type of condition constitutes one of the biggest challenges for organizations, having clear repercussions on the performance and creativity of individuals [17], which can compromise productivity and the quality of the work performed [12,25,34,63,64].

The analysis also showed that teleworking can have mixed impacts [67] regarding workers’ quality of life and health. In more critical circumstances, pathological manifestations tend to be aggravated by the action of a set of other variables, which include work characteristics [76], professional profiles, work quality standards, the support received from employers, personal preferences [9,56,75,78], and family structure [79,81,92]. It is also important to highlight that the impacts of working from home vary according to gender [68]. The analyzes reveal that, especially in relation to women [22], there is an evident negative association between teleworking and mental health [55,58,69,72,78,81] due to different responsibilities and care for children [9,11,56,68,69,71], and the inequalities observed in the execution of domestic tasks [48,79]. Finally, individuals with lower levels of education showed lower tendencies towards psychological problems than those with higher educational levels [54]. These results contradict the ample evidence that low socioeconomic position often appears to be correlated with serious mental health disorders, such as depression and burnout.

The benefits of teleworking are countless [7,10,60]. However, especially in the post-pandemic analyses, the implications resulting from this new work paradigm are clear [91]. In this sense, of the 64 articles reviewed, more than 90% of the studies analyzed the negative impacts of working from home on the physical and mental health of workers. By way of conclusion, working from home can lead to several mental health problems [9], which causes psychological and psychiatric suffering if sufficient working conditions and adequate are not provided [66,71,76]. Given this finding, effective organizational strategies are essential to mitigate adverse effects. In this context, it is necessary to implement psychological support measures, educate on techniques to deal with stress and work overload [10,28], and apply specific guidelines to establish the boundaries between work and personal life [71]. Additionally, it is extremely important to highlight the need for technical and social support as an organizational strategy with the aim of promoting opportunities for interaction between workers based on two-way communication and trust [9,10,65]. To this end, it is vital to use a broad approach and promote adequate emotional support, which involves both individuals and superiors, in order to guarantee the required balance between professional and personal life [31].

## 4. Conclusions

Teleworking as a resource that facilitates the performance of professional functions remotely is not a recent phenomenon, as proven by Mann and Holdsworth [21]. These authors highlighted not only its benefits but also the potential implications for workers’ mental health. The COVID-19 pandemic has profoundly changed working conditions and the way of working in today’s society [79] and has led to significant transformations, namely the intensification of remote work. The literature points out that the pandemic was assumed to be an environmental factor potentially capable of influencing and modifying current work approaches [12]. Today, in a context of increasing adoption of teleworking, several researchers continue to confirm the impacts of this resource on the health of teleworkers. In this sense, it is pertinent to state that, twenty years later, the findings of Mann and Holdsworth remain relevant.

This systematic review offers a comprehensive framework regarding the complex relationship between teleworking and workers’ mental health. From the analyzes carried out, it became clear that teleworking has a dualistic nature. On the one hand, several authors report favorable experiences associated with remote work, e.g., [78,99]. And, on the other hand, other studies do not support the idea that this work pattern leads to permanent positive results [69]. It must be considered that this last scenario could significantly compromise the quality of life of workers, affecting their family and professional lives [11]. The most recent publications have highlighted that teleworking is a major challenge for organizations due to the serious concerns it has raised regarding the workforce and their physical and psychological health. Considering that the workers’ health and mental well-being is a decisive factor for the sustainability, productivity, and efficiency of companies, it is argued that supporting mental health at work should no longer be seen as a secondary option but as a priority for organizations, and even an organizational intervention [99]. The bottom line that can be defined is that there are both positive and negative outcomes of remote working since working from home is an inevitable part of our daily lives. In this sense, it is essential that companies adopt appropriate and effective internal measures, aiming to promote a healthy work climate that supports a positive and satisfactory environment.

## 5. Practical Implications

This study constitutes a relevant contribution to the academic and business worlds, allowing a comprehensive understanding of the phenomenon of teleworking with several practical implications. As a first approach, and considering that teleworking is a permanent reality, it is important to highlight the discussion about the impacts on workers’ mental health. This issue is important, as teleworking (which can also be a hybrid modality) is also a preference for workers. Deepening the understanding of this new work reality and analyzing its implications in the work environment will allow policymakers to implement organizational measures to promote a healthy organizational culture and the development of sustainable management policies that enable the well-being of workers at home, reduce turnover, and sustain efficient and productive teams.

These results may represent a contribution to strategic decision making related to the implementation of teleworking, helping to mitigate the potential risks inherent to this type of work. In this sense, employers must be aware of the challenges of imposing teleworking, particularly with regard to gender equality, considering the disproportional labor differences between men and women. Hence, this study offers relevant strategic content, which allows us to design a path to respond to the psychosocial challenges associated with teleworking. Hence, it is crucial that companies develop a solution that encompasses communication, technical support, and a diversity of tasks. The type of management and leadership exercised are decisive for the correct implementation of solutions that prove to be effective.

Finally, organizations must consider the work needs of teleworkers prior to implementation/imposition in order to positively adapt to the home office environment. Working remotely should not mean a lack of communication, connection, sharing, or mutual support. On the contrary, companies must be able to provide teleworkers with the feeling of continuing to belong to the company. In this context, it is important to ensure a preventive approach to the potential negative effects that teleworking can have on different spheres of an individual’s life.

## 6. Limitations

Despite the obvious contributions, this study presents some limitations that should be addressed in future studies in order to carry out a more complete approach to scientific knowledge.

Firstly, it is important to recognize that this research only included one database, even though Scopus is the largest database of peer-reviewed literature, with bibliometric tools to track, analyze, and visualize the research. Therefore, in future research, it is necessary to use other databases to expand and compare the structure of knowledge regarding teleworking and its effects on workers’ mental health. Studying the impact of the effects of the pandemic can be an ambiguous task, as there are no specific instruments to measure and ensure that the effects actually arise from COVID-19. In this sense, generalizing the results of this recent post-pandemic period may not be representative or offer conclusive evidence regarding the consequences of teleworking for mental health. It is also necessary to highlight that this research does not consider other type of resources (e.g., book chapters). In the future, it is important that scholars apply for these criteria to ensure quality and broader scientific knowledge.

## 7. Future Research Lines

This systematic literature review made it clear that the number of publications on teleworking and its effect on mental health has increased exponentially in recent years. It is undeniable that COVID-19 has worsened both the prevalence of this type of work and the effects it entails. Considering that the incidence of long-term COVID varies from 7.5% to 41% [100], future studies examining these symptoms are essential, particularly with regard to comparisons between the health problems associated with teleworking and the possibility of long-term COVID worsening the set of symptoms. Furthermore, an analysis of specific teleworking interventions and policies that promote the mental health and well-being of workers is essential to ensuring a balance between work and personal life.

In this same framework, the need for in-depth research into the development, manifestation, and prevalence of the mental symptoms that most affect workers are also highlighted. As the indicators of greater suffering, they should be reconsidered when implementing teleworking in a company context. Additionally, post-COVID research may also involve comparative studies between countries that have different work cultures, seeking to study whether, due to different cultures and geographic factors, there are significant differences between them. Considering that teleworking is gaining more and more followers, it is important to evaluate in future research the real benefits of remote working from the perspective of productivity and organizational performance. The approach adopted must identify risk factors that compromise business performance and jeopardize the company’s long-term sustainability. In this sense, based on this systematic review, research must be designed with the ultimate aim of providing both workers and organizations with the relevant knowledge for a positive adaptation to teleworking. On this assumption, and as the implementation of teleworking gains prominence, research in this field will assume a preponderant role in promoting the mental health of workers as well as in the development of support policies aimed at positive and sustainable companies.

## Figures and Tables

**Table 1:** Methodologies used.

Research Method	%	Sample	# of Articles
Qualitative	15.63	University workers	1
		Health care workers	2
		Public workers	2
		General workers	5
Quantitative	71.88	Academic staff and students	5
		Teachers	7
		ICT workers	1
		Public workers	2
		Health care workers	3
		General workers	28
Mix-method	3.13	Teachers	1
		General workers	1
Non-empirical	9.36		6

**Table 2:** Summary of clusters.

Cluster	Keywords	% Articles	Example
1. Work effects on health	Health impact, health status, job satisfaction, occupational health, working conditions, working time, and workplace	40%	Niebuhr, Borle, Börner-Zobel, and Voelter-Mahlknecht (2022). Healthy and happy working from home? Effects of working from home on employee health and job satisfaction [52]
2. Pandemic effects	COVID-19, epidemiology, lockdown, mental health, quality of life, teleworking, and well-being	24%	Xiao, Becerik-Gerber, Lucas and Roll (2021). Impacts of working from home during COVID-19 pandemic on physical and mental well-being of office workstation users [48]
3. Emotional effects	Anxiety, burnout, depression, and work environment	19%	Perelman, Serranheira, Pita Barros, and Laires (2021). Does working at home compromise mental health? A study on European mature adults in COVID times [53]
4. Stress and teleworking	Distress syndrome, mental stress, and psychological distress	17%	De Sio, Cedrone, Nieto, Lapteva, Perri, Greco, Mucci, Pacella, and Buomprisco (2021). Telework and its effects on mental health during the COVID-19 lockdown [54]

## Data Availability

All data generated and analyzed during this review are included in the published review article.
